# Physician's Burnout and the COVID-19 Pandemic—A Nationwide Cross-Sectional Study in Austria

**DOI:** 10.3389/fpsyt.2021.784131

**Published:** 2021-12-07

**Authors:** Ilsemarie Kurzthaler, Georg Kemmler, Bernhard Holzner, Alex Hofer

**Affiliations:** Division of Psychiatry I, Department of Psychiatry, Psychotherapy and Psychosomatics, Medical University Innsbruck, Innsbruck, Austria

**Keywords:** COVID-19 pandemic, burnout, psychological distress, physicians, general practitioners

## Abstract

**Background:** The current study assesses the prevalence of burnout and psychological distress among general practitioners and physicians of various specialities, who are not working in a hospital, during the COVID-19 pandemic. Additionally in this context, contributing factors are registered.

**Materials and Methods:** Burnout and psychological distress were assessed with the Copenhagen Burnout Inventory (CBI) and the Brief Symptom Inventory (BSI-18). A newly developed self-reporting questionnaire was used to evaluate demographic data and pandemic-associated stress factors.

**Results:** 252 general practitioners and 229 private practice physicians provided sufficient responses to the outcome variables for analysis. The prevalence of clinically relevant psychological distress was comparable between groups (12.4 vs. 9.2%). A larger proportion of general practitioners than specialists had intermediate (43.8 vs. 39.9%) or high burnout (26.9 vs. 22.0%) without reaching statistical significance for either category. When combining study participants with intermediate and high levels of burnout, the group difference attained significance (70.7 % vs. 61.9%).

**Conclusion:** Our findings provide evidence that practicing physicians are at high risk of burnout in the context of the pandemic. Being single (standardized beta = 0.134), financial problems (beta = 0.136), and facing violence in patient care (beta = 0.135) were identified as significant predictors for psychological distress. Burnout was predicted by being single (beta = 0.112), financial problems (beta= 0.136), facing violence in patient care (beta = 0.093), stigmatization because of treatment of SARS-CoV-2-positive patients (beta = 0.150), and longer working hours during the pandemic (beta = 0.098).

## Introduction

Even before the COVID-19 pandemic, physician burnout was a topic that was increasingly attracting scientific attention. Numerous studies have reported a greater prevalence of burnout amongst physicians in comparison to individuals in other careers (1), with a reported burnout rate ranging between 30 and 65% across medical specialities, with particular reference to those working on the front line of clinical care, in general internal medicine, and in emergency medicine (2).

Burnout is a psychological syndrome characterized by exhaustion, depersonalization (e.g., cynicism or negativity), and reduced professional efficacy (3). Overall, physician burnout is socio-politically important because it is associated with negative consequences on patient care, the physician workforce, healthcare system costs as well as physicians' own care and safety (4). Additionally, the literature provides indications of a correlation between burnout and an increased use of alcohol and drugs, physical exhaustion, family problems, depression, and suicide, and thus underlines the relevance of the burnout syndrome (5).

In Austria, Mayrhofer reported in 2011 (6) that 54% of the 6,249 Austrian physicians questioned showed burnout symptoms at various levels of severity. Out of those, 14% suffered from symptoms of high intensity. In 2017, Kurzthaler et al. (7) found similar results in our study about the prevalence and the severity of burnout symptoms in a sample of clinical physicians from various disciplines. These results illustrate the extent of burnout among physicians in Austria and emphasize the fact that the presence of burnout in the healthcare profession was already a serious issue before the novel coronavirus disease (COVID-19) was initially identified.

On March 11, 2020, the World Health Organization (8) officially declared the COVID-19 infection a pandemic. Studies carried out on previous outbreaks [severe acute respiratory syndrome (SARS), Middle East respiratory syndrome (MERS), influenza, and Ebola epidemics] already describe signs of severe emotional distress in medical practitioners during outbreaks and face posttraumatic stress disorder (PTSD), depression, anxiety, and burnout after the threat of the outbreak has safely passed (9). However, the COVID-19 pandemic has intensified stressors in healthcare systems worldwide. Along with its high infection and fatality rates, it has generated a universal psychosocial impact by causing economic burden and financial losses (10). Healthcare workers in particular, such as physicians, are being affected during the COVID-19 pandemic by factors such as long working hours, enormous pressure to guard against the extreme danger of infection, shortages of protective equipment, frustration, discrimination, loneliness, and exhaustion (11). Recent literature already confirms that this wide range of occupational and personal stressors results in anxiety and depression and, simultaneously, that stress and anxiety are risk factors for the development of burnout at varying degrees (12). Noticing these scientific findings, one can argue that there will be an increase in the risk of burnout among physicians not only during but also after the COVID-19 pandemic, an escalation that is obviously dangerous especially because it arises from a pre-existing high baseline rate of burnout from before the COVID-19 pandemic.

To our knowledge, there are only a few current pieces of research that give any information about the impact of the COVID-19 pandemic on physician burnout. Wu et al. (13) first explored the prevalence of burnout amongst medical staff in China, when China was the epicenter of the virus. They reported that almost 23% of physicians had felt more burnout compared to the situation before the COVID-19 crisis. A further Italian study conducted by Giusti et al. (14) showed that more than two thirds of participants had reported moderate to severe levels of emotional exhaustion and reduced professional efficacy, and more than a quarter of the sample reported moderate to severe levels of depersonalization. At the same time, this same level of physician burnout (on average, 76%) was reported in a Romanian study by Dimitriu et al. (15). Here, the authors noted that this level of burnout was ‘superior to [that found in] studies conducted in normal periods'. The main reported predictors of burnout during the current pandemic consist of occupational factors such as place of work and increased working hours as well as the factor of female gender (5).

The aim of the current study was to evaluate the extent of burnout among general practitioners and physicians of various specialities who are not working in a hospital (referred as specialists in the rest of the manuscript) during the COVID-19 pandemic in Austria with respect to psychological distress and its underlining variable stress factors. Additionally, we focussed on the consideration of potential contributing factors and their role in physician burnout.

## Materials and Methods

### Study Population and Study Design

Together with the Austrian Medical Association, we conducted a cross-sectional, anonymous, self-administered, web-based study to investigate the prevalence of burnout among general practitioners and specialists during the COVID-19 pandemic. All based in Austria, practicing physicians received an email from their local medical association giving them prior notice and a study invitation together with the link and quick response (QR) code to access the electronic questionnaire. The questionnaire as well as the responses were received anonymously. The study was online from August 17th to November 22nd, 2020. Overall, 481 participants' responses were analyzed. The Ethics Committee of the Medical University Innsbruck, Austria approved the study protocol and procedures of informed consent. Participants had to answer a yes/no question to confirm their willingness to participate voluntarily. Only after confirmation of that question was the participant able to complete the self-reporting questionnaire.

### Sociodemographic and COVID-19 Related Variables

In the first part of the survey, sociodemographic data were collected, including age, gender, partnership status, etc. as well as the duration of professional experience, and the location of surgery. In addition, some COVID-19 related data were collected, e.g., whether participants or their relatives had been tested positive for SARS-CoV-2, whether they had treated SARS-CoV-2-positive patients, and whether they had therefore experienced stigmatization. In addition, study participants were asked whether they felt exposed to more violence in patient care during the pandemic. An additional pool of data collected regarded the perception and acceptance of COVID-19-related measures and whether study participants had sufficient protective equipment to implement them.

### Instruments

#### Questionnaire to Evaluate Demographic Data and Pandemic-Associated Stress Factors

We used a newly developed self-reporting questionnaire to record personal characteristics and COVID-19-related stress. The questionnaire assessed both individual and work-related strains, socioeconomic impacts (both before and during the pandemic), influence of workplace location (e.g., urban, rural, in a quarantine zone), rational understanding for preventive measures, and satisfaction with provided protective equipment as well as the information from public authorities. Additionally, physicians' fear of or concern about becoming infected with COVID-19, or transmitting it to their relatives, and affliction regarding stigma were evaluated. Most of the items had a simple binary response format (yes/no). Only a few items were open questions requiring free-text answers.

#### Copenhagen Burnout Inventory

The first and most commonly used instrument to evaluate burnout in different demographic and professional populations was the Maslach Burnout Inventory (MBI) (16). It became the gold standard metric of burnout. However, in recent years, authors began to criticize the unbalance and dubiety between the three dimensions of burnout evaluated by the MBI (17, 18). To cope with these shortcomings we used the newly created psychometrically valid Copenhagen Burnout Inventory (CBI) (16), which consists of three scales measuring personal burnout, work-related burnout, and client-related burnout through a 19-item survey. It allows clinicians and researchers to obtain one simple global burnout index to facilitate a clear and easy evaluation of the extent of professional burnout (19).

In the CBI, the core of burnout is fatigue and exhaustion. This is analogous with a more recent definition of burnout, which specifies this syndrome as a state of physical, emotional, and mental exhaustion that results from long-term involvement in work situations that are emotionally demanding (20). Personal burnout is the extent of physical and psychological fatigue and exhaustion experienced by the person. The work-related subscale measures the degree of physical and psychological fatigue and exhaustion that a person recognizes as work-related. Finally, the patient-related subscale shows the dimension of physical and psychological fatigue and exhaustion that a person classifies as being attributed to the work done with patients. Items within the subscales are averaged, with possible score ranges for all scales of 0–100 and higher scores indicating a higher degree of burnout. In the literature, scores of 25 or lower, 25 to 50, and higher than 50 categorize low, intermediate, and high burnout, respectively (19). Internal consistency (Cronbach's α) was excellently high with α = 0.93.

#### Brief Symptom Inventory

The BSI-18 (21) is a short form consisting of 18 items taken from the Symptom Checklist (SCL)-90-R. The BSI-18 assesses three six-item symptom scales—somatization, depression, and anxiety—and includes the global scale Global Severity Index (GSI). BSI-18 scores are calculated by sum scores. The total score therefore ranges from 0 to 72. The translation from raw scores into T-scores is based on gender-specific normative data from the general healthy population. As recommended by the authors of the instrument, GSI T-scores ≥ 63 were considered as clinically relevant psychological distress. Normative data for the BSI-18 derive from a sample of community-dwelling adults all employed by a single corporation in the United States, and its psychometric properties have been confirmed in a representative sample from Germany (22). The BSI-18 case-standard is identical to the standard used with the SCL-90-R. Internal consistency of BSI-18 scales as quantified by Cronbach's α ranged from 0.71 to 0.84, which is similar to the internal consistency reliabilities reported in a large sample of survivors (23), where α ranged from 0.75 to 0.88. For our sample we report a high internal consistency with α = 0.90.

### Statistical Methods

IBM SPSS 26 was used for statistical analysis. For comparisons between the two physician groups regarding sociodemographic and COVID-19-related variables, Fisher's exact test, the Chi-squared test, and the Mann-Whitney U test were applied, depending on the variable type (dichotomous, categorical, or metric variables, respectively, where the majority of the metric variables had non-normal distribution). For comparisons between the situations before and during the COVID-19 pandemic, the McNemar test was used for dichotomous and Wilcoxon's matched-pairs test for metric variables with non-normal distribution. Spearman rank correlations were determined to analyze the relationship between sociodemographic and COVID-19-related variables on the one hand and burnout (CBI) as well as psychological distress (BSI-18) on the other. The size of the correlation coefficient can be interpreted as follows: *r* < 0.10 no correlation; *r* = 0.10–0.29 low correlation; *r* = 0.30–0.49 moderate correlation; and *r* ≥ 0.50 high correlation (24).

Multiple linear regression analyses were performed to investigate possible effects of demographic and COVID-19-related variables on the BSI and CBI scores of physicians. Only those COVID-19-related variables that had attained significance in the correlation analysis were considered in the regression analyses. Sociodemographic variables were entered first to adjust for potentially confounding effects. The other independent variables were entered by stepwise forward selection at a 0.05 level of significance. R squared was reported as an overall measure of goodness of fit.

## Results

### Sociodemographic Variables

In this nationwide online study, we obtained useable responses from 252 general practitioners and 229 physicians of various specialities. This accounts for ~5% of all Austrian general practitioners and 3.5% of all private practice specialists (25). The mean age of the specialist sample was higher than that of the practitioner sample. Concerning the private household members and the number of children, values were significantly higher in the group of general practitioners than in the group of specialists. The specialists' surgeries were located in urban areas, in a tourist location, or in an area strongly affected by COVID-19 more often than the general practitioners' surgeries were. Details are shown in Table 1.

**Table 1:** Sociodemographic variables (*N* = 481).

**Variable**	**GP (*N* = 252)**	**Specialists (*N* = 229)**	**Statistics^a^**	***p*** **-value**
**Gender**				
Male	127 (50.4%)	106 (46.3%)	χ^2^ = 2.86; df = 2	0.239
Female	125 (49.6%)	121 (52.8%)		
Others	0 (0.0%)	2 (0.9%)		
Age (years)	51.9 ± 8.4	53.5 ± 7.7	*U* = 25717; *Z* = 1.99	0.046
**Relationship**				
Fixed partnership	231 (91.7%)	207 (90.4%)	χ^2^ = 0.24; df = 1	0.635
Single	21 (8.3%)	22 (9.6%)		
People in household	3.1 ± 1.2	2.8 ± 0.9	*U* = 24038.5; *Z* = 3.12	0.002
Children	1.2 ± 1.1	1.0 ± 1.0	*U* = 24853; *Z* = 2.62	0.009
Duration of professional experience (years)	15.3 ± 10.4	14.1 ± 9.6	*U* = 27040; *Z* = 1.04	0.298
Surgery in a rural area	155 (61.8%)	74 (32.6%)	χ^2^ = 40.6; df = 1	<0.001
Surgery in an urban area	110 (43.7%)	184 (80.6%)	χ^2^ = 68.0; df = 1	<0.001
Surgery in tourist location	110 (43.7%)	127 (55.5%)	χ^2^ = 6.69; df = 1	0.011
Surgery in an area strongly affected by COVID-19	74 (30.5%)	94 (43.3%)	χ^2^ = 8.18; df = 1	0.005

*mean ± standard deviation or N (%)*.

a *Metric variables were analyzed with the Mann–Whitney U-test, categorical variables with the Chi-squared test; Fisher's exact test was used for 2 × 2 tables*.

*GP, General practitioners; df, degrees of freedom*.

### Work-Related Aspects

Details of work-related aspects before and during the COVID-19 pandemic are shown in Table 2. In the group of general practitioners, the weekly working time remained stable, whereas in the specialist group the working hours declined by ~10 h per week from 2019 to 2020 (before vs. during the pandemic). The number of patients seen per quarter decreased significantly in both groups, both in the first and second quarter of the respective years (2019 vs. 2020). In each of the groups, the percentage of respondents reporting financial problems increased considerably, from below 10% to about 23%.

**Table 2:** Work-related aspects before and during the COVID-19 pandemic.

**Variable**	**GP (*N* = 252)**	**Specialists (*N* = 229)**
Working hours/week before COVID-19	43.1 ± 13.1	38.2 ± 12.6^§^
Working hours/week during COVID-19	42.1 ± 17.7	28.6 ± 14.2↓
Patients per quarter 2019 (average)	1417 ± 795	986 ± 813^§^
Number of patients Q1/ 2019	1457 ± 806	1011 ± 934^§^
Number of patients Q1/ 2020	1366 ± 702↓	890 ± 808↓^§^
Number of patients Q2/ 2019	1384 ± 828	969 ± 908^§^
Number of patients Q2/ 2020	1127 ± 615↓	758 ± 723↓^§^
Financial problems before COVID-19	17 (7.0%)	8 (3.7%)
Financial problems during COVID-19	56 (23.0%)↑	50 (22.9%)↑

*Mean ± standard deviation or N (%)*.

§ *Significantly lower than in the GP group (Mann–Whitney U-test, |Z| > 4.0, p < 0.001)*.

*↓Significantly lower than before the pandemic (Wilcoxon matched-pairs test, |Z| > 5.0, p < 0.001)*.

*↑Significantly higher than before the pandemic (McNemar test, Chi-squared > 33.0, p <0.001)*.

*GP, General practitioners; Q, quarter*.

### Further COVID-19-Related Variables

Table 3 shows a comparison of various other COVID-19-related variables, general practitioners were confronted with a significantly higher percentage of SARS-CoV-2-positive patients and were faced with significantly more deaths due to COVID-19 than the specialists were. Moreover, a significantly larger proportion of general practitioners had insufficient supplies of equipment to implement prescribed protective measures as compared to specialists. The COVID-19-related measures were considered to be justified by a higher percentage of specialists than general practitioners. Moreover, specialists generally felt better informed about the COVID-19-related measures than general practitioners did.

**Table 3:** Further COVID-19-related variables.

	**GP (*****N*** **=** **252)**		**Specialists (*****N*** **=** **229)**	
	***n***	**%**	***n***	**%**
Belonging to COVID-19-risk group	53	21.8%	52	23.9%
SARS-CoV-2 test performed	122	50.2%	92	42.2%
Positive SARS-CoV-2 test result	8/122	6.6%	3/92	3.3%
Suspected case	34	14.5%	22	10.2%
Quarantined	43	17.7%	27	12.4%
Positive SARS-CoV-2 test result in relatives	19	7.8%	10	4.6%
Relatives who died from COVID-19	2	0.8%	0	0.0%
Treatment of SARS-CoV-2-positive patients^a^	199	81.9%	108	49.5%
Faced with patients who died from COVID-19^b^	41	16.9%	14	6.5%
Faced with more violence in patient care during the pandemic	19	7.8%	23	10.6%
COVID-19-related measures considered adequate^c^	164	67.5%	168	77.1%
Closure of practice ordered by authorities	17	7.0%	10	4.6%
Sufficiently informed about COVID-19-related measures^d^	116	47.7%	167	76.6%
Exchange of information with colleagues	135	55.6%	114	52.3%
Sufficient protective equipment to implement measures^e^	68	28.0%	109	50.0%
Stigmatization because of treatment of SARS-CoV-2-positive patients	85	35.0%	61	28.1%

a *Significantly higher in the GP group than in the specialist group, χ^2^ = 54.0, p < 0.001*.

b *Significantly higher in the GP group than in the specialist group, χ^2^ = 11.9, p < 0.001*.

c *Significantly higher in the specialist group than in the GP group, χ^2^ = 5.52, p = 0.022*.

d *Significantly higher in the specialist group than in the GP group, χ^2^ = 40.4, p < 0.001*.

e *Significantly higher in the specialist group than in the GP group, χ^2^ = 23.6, p < 0.001*.

*GP, General practitioners*.

### Psychological Distress and Burnout

As Table 4 shows, the two physician groups did not show significant differences in psychological distress, neither in the BSI-18 total scores nor in the prevalence of clinically relevant psychological distress (GSI T), showing values near 10% in both groups. Regarding burnout, however, general practitioners exhibited significantly higher scores in personal and client-related burnout as well as in the CBI total score. In line with the classification of burnout defined above, a larger proportion of general practitioners than specialists presented intermediate burnout (44 vs. 40%), and the same was true for high burnout (27 vs. 22%), without reaching statistical significance for either category. However, when combining intermediate and high levels of burnout, the difference attained significance (70.7 vs. 61.9%, *p* = 0.047).

**Table 4:** Burnout and psychological distress.

	**GP**			**Specialists**			**Comparison**		
	**Mean**	***SD***	***N***	**Mean**	***SD***	***N***	***d***	**Statistic**	***p*** **-value**
**Burnout (CBI)**									
Personal burnout	41.3↑	20.1	242	37.1	21.0	218	0.20	*Z* = 2.38	0.017
Work-related burnout	35.6	19.6	242	32.6	20.6	218	0.15	*Z* = 1.70	0.088
Client-related burnout	36.6↑	19.6	242	32.0	20.3	218	0.23	*Z* = 2.50	0.012
CBI total score	37.7↑	18.2	242	33.8	19.5	218	0.21	*Z* = 2.29	0.022
Intermediate burnout (CBI total 25–50)	43.8%			39.9%				χ^2^ = 3.93	0.047
High burnout (CBI total > 50)	26.9%			22.0%					
**Psychological distress**									
BSI-18 total (range 0–72)	7.02	7.74	241	6.43	6.46	218	0.08	*Z* = 0.47	0.636
Clinically relevant psychological distress (T-score ≥ 63)	12.4%			9.2%				χ^2^ = 1.26	0.261

*↑Significantly higher than in the specialist group*.

*GP, General practitioners*.

*Burnout (CBI): Items within the subscale are averaged, with possible score ranges for all scales of 0–100 and higher scores indicating a higher degree of burnout*.

### Correlation Analyses

Findings of the correlation analyses are shown in the Appendix Table A1. Regarding sociodemographic, a younger age and the female gender were associated with higher levels of personal and work-related burnout. Single people, in contrast to those with a fixed partner, tended to have higher levels of personal burnout and psychological distress. Among work-related and COVID-19-related variables, the number of working hours in 2019 and an increase in working time from 2019 to 2020 were associated with higher levels of burnout. The latter variable also correlated with higher scores of psychological distress. Financial problems before and during the pandemic correlated with increased levels of both burnout and psychological distress. Further details can be found in Appendix Table A1. Separate correlation analyses for the two physician groups generally yielded similar findings.

### Findings of Regression Analyses

Results of regression analyses for burnout are shown in Table 5A. As the findings for the individual burnout subscales were very similar, we confined this analysis to the CBI total score. The following variables were identified as significant predictors of burnout: being single, financial problems experienced during COVID-19 as well as those already experienced before, stigmatization because of treatment of SARS-CoV-2-positive patients, facing violence in patient care, and longer working hours during the pandemic. Separate regression analyses for the two groups yielded generally similar findings.

**Table 5:** Linear Regression—Burnout (CBI total score) **(A)** and psychological distress (BSI-18 total score) **(B)**.

	**Independent variables**	**Beta**	**S.E. (beta)**	**Standardized beta**	***t***	***p*** **-value**
**Burnout^a^**	**Sociodemographics (included as potential confounders)**					
	Age	0.005	0.074	0.003	0.067	0.947
	Gender female (vs. male)	1.672	1.181	−0.065	1.416	0.157
	**Significant predictors**					
	Single (vs. fixed partnership)	5.040	2.020	0.112	2.495	0.013
	Working hours during pandemic	0.074	0.035	0.098	2.138	0.033
	Financial problems before the pandemic	5.783	2.719	0.101	2.127	0.034
	Financial problems during the pandemic	4.207	1.485	0.136	2.833	0.005
	Stigmatization because of treatment of SARS-CoV-2-positive patients	4.218	1.298	0.150	3.249	0.001
	Faced with more violence in patient care during the pandemic	4.205	2.043	0.093	2.058	0.040
**Psychological distress^b^**	**Sociodemographics (included as potential confounders)**					
	Age	−0.003	0.002	−0.063	−1.399	0.163
	Gender female (vs. male)	0.014	0.036	0.017	0.375	0.708
	**Significant predictors**					
	Single (vs. fixed partnership)	0.192	0.064	0.134	3.026	0.003
	Financial problems before the pandemic	0.345	0.083	0.196	4.152	<0.001
	Financial problems during the pandemic	0.130	0.046	0.136	2.828	0.005
	Faced with more violence in patient care during the pandemic	0.186	0.062	0.135	3.017	0.003

a *Model information: adjusted R^2^ = 0.115, F = 8.34, d.f. = 8, and p < 0.001*.

b *Model information: adjusted R^2^ = 0.182, F = 14.53, d.f. = 7, and p < 0.001*.

*BSI-18, Brief Symptom Inventory; CBI, Copenhagen Burnout Inventory*.

*(A) The following variable was not included in the regression model as it did not attain statistical significance: change in working hours (during COVID-19 vs. before)*.

*(B) The following variables were not included in the regression model as they did not attain statistical significance: change in working hours (during COVID-19 vs. before), number of patients in the individual quarters (Q1/2019, Q1/2020, Q2/2019, Q2/2020), and stigmatization because of treatment of SARS-CoV-2-positive patients*.

Findings of the regression analysis for psychological distress are summarized in Table 5B. The following variables emerged as significant predictors of psychological distress: being single, financial problems during COVID-19 as well as those already experienced before, and facing violence in patient care. Separate regression analyses for the two physician groups gave rise to similar findings.

## Discussion

There is a consensus in all the relevant literature that healthcare professionals are at an increased risk of high levels of stress and burnout (26), which could have long-term psychological implications. In this national online survey, we found large proportions of physicians experiencing symptoms of burnout (general practitioners: 70%; specialists: 62%) and psychological distress (general practitioners: 12%; specialists: 9%) during the COVID-19 pandemic. Although psychological well-being was the main question, our research additionally identified factors contributing to these outcomes. We identified sociodemographic as well as work-related and specific COVID-19-related variables as risk factors that can result in physicians' impaired mental health.

Altogether, our results show a substantial increase in burnout rates compared to about 30% described for Austrian physicians in earlier studies before the COVID-19 outbreak (6, 7). Combining intermediate and high levels of burnout, the rate among general practitioners and specialists participating in this study amounted to 70.7 and 61.9%, respectively. This is an alarmingly high number and points to a particular burden in general practitioners. Compared to specialists, their workload remained unchanged during the pandemic despite a lower patient turnover. In addition, they reported significantly more frequently to be involved in the treatment of SARS-CoV-2-positive patients and/or faced with patients who died from COVID-19, to be not well informed about COVID-19-related measures, and to lack sufficient protective equipment. Among other factors, physician burnout has previously been shown to be related to lack of autonomy/control of work schedule, long work hours, financial issues, and inefficient and/or hostile work environments (27, 28). Accordingly, our finding of higher burnout rates in the general practitioner sample is not surprising.

Clinically relevant psychological distress as assessed by the BSI-18 showed comparable values of nearly 10% in both groups. Compared to the high proportion of physicians with burnout this percentage seems relatively low at first sight. One has to consider, however, that although psychological distress does not indicate mental disease but the presence of clinically relevant subjective impairment, burnout and the symptoms captured by the BSI-18 (somatization, depression, and anxiety) can be comorbid. While the society recognizes health care workers' tremendous stressors, the potential for more serious mental health crises among that profession has not received enough attention (29). Offering programs for preventive health maintenance making physicians aware of their affective response to external events like the current pandemic and appropriate mental health care is necessary to counteract psychological distress and burnout in this group. Our findings may indicate that study participants had a notable burnout symptomatology in the form of fatigue and exhaustion in the occupational context, but in a large part, they may have been able to cope, meaning that they protected themselves from developing clinically relevant psychological distress. This hypothesis cannot be tested by our data since we did not investigate coping mechanisms, however, the presence of burnout symptoms has been suggested be a risk factor for the development of clinically relevant psychological distress/mental illness (30) and accordingly, further longitudinal studies are needed to clarify this issue.

In line with previous investigations (26, 31), we detected several associations between burnout and sociodemographic, health- and work-related as well as COVID-19-related factors, even if the findings of correlation analyses were weak. However, the subsequently passed regression analyses showed single life, existing financial problems, stigmatization because of treatment of SARS-CoV-2-positive patients, and facing increased violence in patient care as predictors of both burnout and psychological distress. These findings largely corroborate those of earlier studies from other countries (32). On the other hand and contrary to previous reports, younger age and female sex were neither predictor variables for burnout nor for psychological distress among our sample. However, with regards to age one has to consider that study participants' mean age was above 50 and that the mean duration of self-employed professional experience amounted to ~15 years, whereas poor psychological outcomes have mainly been reported in young physicians at more junior career stages (32). Accordingly, our sample is not entirely comparable with others. Similarly, people with a higher burden or conversely, those with a lower burden may have been more likely to participate in the study and accordingly, a sample bias has to be taken into account. One can hypothesize that this and the fact that we exclusively investigated private practice physicians but not employed physicians may have concealed possible gender differences. However, this issue cannot be addressed by our data.

Remarkably, among both physician groups a decline in the number of patients with a medical condition showed a negative association with burnout and psychological distress. Quite to the contrary, one could assume that fewer patients to medicate would lead to a smaller workload with a reduction of psychological distress and burnout symptomatology. However, physicians probably connect a lower patient turnover with a fall in income that may initiate financial problems. Milch et al. (33), for instance, reported increased financial difficulties and financial loss in a breast imaging community during the COVID-19 pandemic and described them *per se* as linked with higher levels of psychological distress. This corroborates our finding of a positive association between financial problems both before and during the pandemic and psychological distress/burnout. Importantly, in comparison with the previous year's period, the percentage of physicians reporting financial problems during the pandemic tripled among general practitioners and increased six-fold in specialists. It remains to be seen whether this percentage normalizes in the course of the pandemic and whether this may subsequently be associated with a reduction in psychological distress and burnout

To add to the many challenges connected to the COVID-19 pandemic, we are seeing a worrying increase in violence against healthcare workers globally. Those attacks from patients or their relatives have been reported to often originate from the health workers' efforts to implement essential—but unpopular—COVID-19 prevention and control measures (e.g., placing a family member in a quarantine or isolation facility, or not allowing the family to attend to an infected loved one) (34, 35). This response is likely to exacerbate the already extraordinary COVID-19-related stress and burnout that healthcare workers and their families are experiencing in this pandemic. It is therefore not surprising that in the current study more violence in patient care was positively correlated with psychological distress and burnout among the general practitioners as well as among the specialists. Although generally weak, the lowest correlation was detected between the exposition to more violence in patient care and personal burnout, which is intuitively plausible

To the best of our knowledge, this is the first nationwide study conducted in Austria that investigated psychological distress and burnout among general practitioners and specialists during the COVID-19 pandemic. Notwithstanding the implications of our findings, there are a number of limitations, including the small sample size that should be considered. As mentioned above, our findings refer to a minority of Austrian general practitioners and specialists and accordingly, a sample bias has to be taken into account, which limits the generalizability of the obtained results. Those who did not respond may very well-represent those who are experiencing the most psychological distress/burnout. Secondly, the information obtained was obviously self-reported, which can result in social desirability bias. Thirdly, our participant population focused solely on general practitioners and private practice specialists and it remains to be seen if our findings can be repeated in employed physicians. Lastly, it is important to emphasize that during most time of our survey, general recommendations like the mandatory use of protective mouth/nose masks, distance keeping, and vigilant hand washing applied, whereas stricter measures were imposed by the Austrian government at the beginning of November, 2020. They were associated with a number of confinements, e.g., travel restrictions, school and University closure, closure of non-essential retail and commercial establishments, etc. Longitudinal studies are clearly needed to determine whether psychological distress/burnout in physicians changes as the pandemic continues. Notwithstanding these limitations, our study shows that during the first months of the pandemic practicing physicians were at high risk of burnout.

## Conclusion

Our findings provide evidence that practicing physicians are at high risk of burnout in the context of the pandemic. Being single, financial problems, and facing violence in patient care were identified as significant predictors of burnout and psychological distress. Furthermore, burnout was predicted by stigmatization because of treatment of SARS-CoV-2-positive patients and by longer working hours during the pandemic.

### Recommendations

As physician burnout impairs performance and quality of professional services with consequences for physicians, healthcare organizations, and patient outcomes, health systems are prompted to prioritize physicians' health and well-being both during and after the COVID-19 pandemic.

## Data Availability Statement

The raw data supporting the conclusions of this article will be made available by the authors, without undue reservation.

## Ethics Statement

The studies involving human participants were reviewed and approved by Ethikkommission Innsbruck. The patients/participants provided their online informed consent to participate in this study.

## Author Contributions

IK and AH were responsible for draft writing and came up with the idea of producing this study, and also wrote the final manuscript. GK and BH took part in data collection, gave suggestion for data analysis, and assisted in transferring raw data into statistical software. All authors contributed to and approved the final report.

## Funding

This work was supported by the Austrian Medical Association. The funder had no role in the design or conduct of the study; the data collection, management, analysis, or data interpretation; the preparation, review, or approval of the manuscript; or the decision to submit the manuscript for publication.

## Conflict of Interest

The authors declare that the research was conducted in the absence of any commercial or financial relationships that could be construed as a potential conflict of interest.

## Publisher's Note

All claims expressed in this article are solely those of the authors and do not necessarily represent those of their affiliated organizations, or those of the publisher, the editors and the reviewers. Any product that may be evaluated in this article, or claim that may be made by its manufacturer, is not guaranteed or endorsed by the publisher.
