# Supplementary material for: Physician's Burnout and the COVID-19 Pandemic—A Nationwide Cross-Sectional Study in Austria
Source: Front Psychiatry. 2021 Dec 7;12:784131. doi: 10.3389/fpsyt.2021.784131 (PMC8688354; doi:10.3389/fpsyt.2021.784131)
Supplement: Supplementary file 1 [file Table_1.DOCX]

**Appendix:**

**Table A1: Associations of sociodemographic and COVID-19-related variables with burnout (CBI) and psychological distress (BSI-18) – findings of Spearman rank correlation analysis^a^**

|  | CBI  Personal burnout | CBI Work-related burnout | CBI Client-related burnout | CBI total burnout | Psychological distress (BSI total score) |
| --- | --- | --- | --- | --- | --- |
| Age | -0.149** | -0.108* | 0.015 | -0.090 | -0.115* |
| Gender (male vs female) | 0.178** | 0.112* | 0.021 | 0.117* | 0.059 |
| Relationship (single vs. fixed partnership) | 0.114* | 0.090 | 0.076 | -0.104* | 0.153** |
| Working hours during COVID-19 | 0.116* | 0.103* | 0.103* | 0.115* | 0.053 |
| Change in working hours (during COVID-19 vs. before) | 0.215** | 0.176** | 0.151** | 0.193** | 0.156** |
| Number of patients Q1/2019 | -0.054 | -0.028 | 0.016 | -0.024 | -0.102* |
| Number of patients Q1/2020 | -0.047 | -0.023 | 0.034 | -0.014 | -0.108* |
| Number of patients Q2/2019 | -0.091 | -0.072 | -0.028 | -0.069 | -0.126** |
| Number of patients Q2/2020 | -0.056 | -0.033 | 0.017 | -0.027 | -0.093 |
| Financial problems before COVID-19 | 0.214** | 0.193** | 0.150** | 0.201** | 0.222** |
| Financial problems during COVID-19 | 0.266** | 0.233** | 0.174** | 0.244** | 0.268** |
| Patients who died from COVID-19 | 0.068 | 0.110* | 0.056 | 0.088 | 0.051 |
| Faced with more violence in patient care during the pandemic | 0.120* | 0.175** | 0.171** | 0.166* | -0.147** |
| Stigmatisation because of COVID-19 patients | 0.196** | 0.194** | 0.182** | 0.201** | 0.274** |

* p < 0.05 ** p < 0.01

^a^ The following variables did not correlate significantly with burnout or psychological distress and were not included in the table: location of surgery (rural, urban, areas highly affected by COVID-19), positive COVID test, quarantined, patients testing COVID-19 positive, closure of practice ordered by authorities.
